# A Case of Scarlatina

**Published:** 1849-11

**Authors:** William Matthews

**Affiliations:** Eberle, Ind.


### ARTICLE II.

*A Case of Scarlatina.*—By WILLIAM MATTHEWS, M. D., of Eberle, Ind.

MESSRS. EDITORS:—

I send you the subjoined history of a case of Scarlatina, which, although it terminated unfortunately, will afford additional evidence to that already accumulated, in favor of the curative powers of Iodine in Hydrocephalus.

A child, eighteen months of age, of rather feeble constitution and strumous habit, was exposed to the contagion of Measles about the 15th of April, and in two or three days thereafter, Scarlet Fever broke out in the family to which it belonged, thus exposing it to the contagion of this dreadful malady. On the 22nd the child was seized with vomiting, followed by a burning fever, and accompanied with the eruption peculiar to Scarlet Fever. The anginose type was determined, and the various glands of the cervical region, the tonsils and parotids were intumescent.

The practice adopted was that usually prescribed by the profession in this variety of Scarlet Fever, and on the fith day it was believed that recovery would speedily ensue. But at this time the eruption became more distinct, was less uniform, and in some measure crescent shaped, closely resembling the rubeolous efflorescence. And, moreover, catarrhal symptoms were present. The diagnosis was Measles---but it yet remains uncertain, (as no other member of the family contracted Measles,) whether the diagnosis was correct or not. At this time the child's strength began to give way, and I was compelled to prescribe ammonia and wine, and its bowels were from time to time evacuated by enemata. Under this plan of treatment I again was led to believe that improvement was going on---the eruption, meantime, standing fully out.

But at the very moment that I was anxiously expecting convalescence, head symptoms began to declare themselves. Believing that these were due to gastro-enteric irritation and general nervous prostration, cold lotions were directed to be kept constantly applied to the head, and Dover's Powder was watchfully prescribed as an internal medicine. The patient, however, gradually grew worse and worse, and at the end of twenty-four hours all hope for its recovery seemed vain. The head was abnormally hot, and coma, strabismus and partial paralysis were the symptoms upon which the diagnosis was predicated---the coma and strabismus being the first signs of the approach of the secondary inflammation.

As a deriner resort at this critical period, one drachm of the saturated solution of Iodine in alcohol, fifty grains of Iodide of Potassium, and three-fourths of an ounce of water formed a solution, of which forty drops were given every three hours until more than half of the contents of the vial were given. In twelve hours the head symptoms were beginning to disappear; pretty profuse diuresis took place, and the medicine was directed to be continued in reduced doses, which being followed up, in the course of two days from the commencement, the whole amount was exhausted. By this time every head symptom had disappeared. Some additional gastro-enteric irritation followed, but emollient fomentations had the effect of removing this, and the patient once more was on the point of convalescence.

Unfortunately, however, the swelling of the cervical glands, just at this period, began to increase; the surrounding parts became implicated in the intumescence, and the functions of the wind-pipe and œsophagus were being interfered with.--- Poultices were applied to the inflamed parts, and wine I was compelled to allow for the purpose of supporting the flagging powers, and hastening the maturating process, which was now evidently approaching. But in spite of nutritious soups, wine, etc., the child continued to sink, and on the 16th of May it died of inanition, before fluctuation could be detected in the inflamed cervical glands---swallowing having, in a great measure, been prevented for several days previous to death.

